# Tricaesium dimolybdate(VI) bromide

**DOI:** 10.1107/S1600536809046297

**Published:** 2009-11-07

**Authors:** Anna S. Pakhomova, Sergey V. Krivovichev

**Affiliations:** a St Petersburg State University, Universitetskaya nab. 7/9, 199034 St Petersburg, Russian Federation

## Abstract

The title compound, Cs_3_(Mo_2_O_7_)Br, was synthesized by the reaction of CsNO_3_, MoO_3_ and 1-ethyl-3-methyl­imidazolium bromide. Its crystal structure is isotypic with K_3_(Mo_2_O_7_)Br and contains (MoO_4_)^2−^ tetra­hedra which share an O atom to produce a [Mo_2_O_7_]^2−^ dimolybdate(VI) anion with a linear bridging angle and 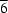 *m*2 symmetry. The anions are linked by Cs atoms (site symmetry 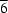 *m*2), forming sheets parallel to (001). Br atoms (site symmetry 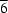 *m*2) are also part of this layer. Another type of Cs atom (3*m* site symmetry) is located in the inter­layer space and connects the layers *via* Cs—O and Cs—Br inter­actions into a three-dimensional array.

## Related literature

For the isotypic compound K_3_(Mo_2_O_7_)Br, see: Becher & Fenske (1978 ▶). For dimolybdates with similar condensed anions made up of MoO_4_ tetra­hedra, see: Ce_2_(MoO_4_)_2_(Mo_2_O_7_) (Fallon & Gatehouse, 1982 ▶); Mg_2_Mo_2_O_7_ (Stadnicka *et al.*, 1977 ▶).

## Experimental

### 

#### Crystal data

Cs_3_(Mo_2_O_7_)Br
*M* *_r_* = 782.52
Hexagonal, 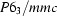

*a* = 6.3993 (5) Å
*c* = 16.4870 (15) Å
*V* = 584.71 (8) Å^3^
*Z* = 2
Mo *K*α radiation
μ = 14.77 mm^−1^
*T* = 293 K
0.15 × 0.15 × 0.05 mm

#### Data collection

Stoe IPDS-2 diffractometer
Absorption correction: integration (*X-RED* and *X-SHAPE*; Stoe, 2005 ▶) *T*_min_ = 0.153, *T*_max_ = 0.532
5224 measured reflections
344 independent reflections
338 reflections with *I* > 2σ(*I*)
*R*_int_ = 0.044

#### Refinement

*R*[*F* ^2^ > 2σ(*F* ^2^)] = 0.025
*wR*(*F* ^2^) = 0.062
*S* = 1.18
344 reflections
20 parameters
Δρ_max_ = 0.60 e Å^−3^
Δρ_min_ = −1.21 e Å^−3^

### 

Data collection: *X-AREA* (Stoe, 2007 ▶); cell refinement: *X-AREA*; data reduction: *X-RED* (Stoe, 2005 ▶); program(s) used to solve structure: *SHELXS97* (Sheldrick, 2008 ▶); program(s) used to refine structure: *SHELXL97* (Sheldrick, 2008 ▶); molecular graphics: *ATOMS* (Dowty, 1999 ▶); software used to prepare material for publication: *publCIF* (Westrip, 2009 ▶).

## Supplementary Material

Crystal structure: contains datablocks global, I. DOI: 10.1107/S1600536809046297/wm2275sup1.cif

Structure factors: contains datablocks I. DOI: 10.1107/S1600536809046297/wm2275Isup2.hkl

Additional supplementary materials:  crystallographic information; 3D view; checkCIF report

## Figures and Tables

**Table 1:** Selected bond lengths (Å)

Mo—O1	1.725 (4)
Mo—O2	1.8764 (7)

**Fig. 1.**
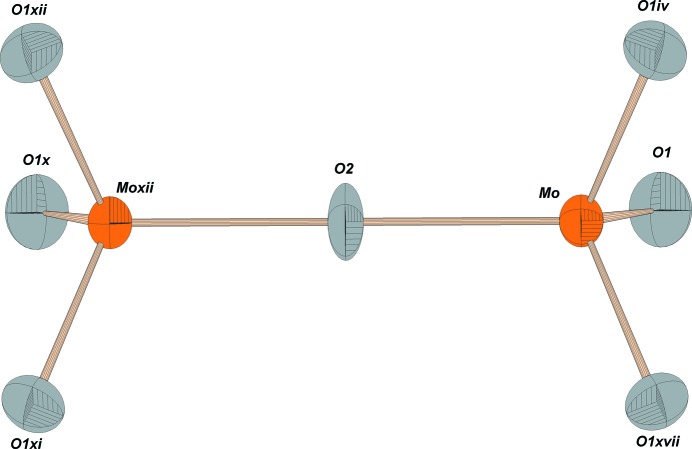
View of the linear [Mo2O7]2- anion. Ellipsoids are drawn at the 50% probability level. [Symmetry codes: (iv) -y+1, x-y-1, z; (x) -x+y+2, -x, -z-1/2; (xi) -y, x-y-2, -z-1/2; (xii) x, y, -z-1/2; (xvii) -x+y+2, -x+1, z.]

**Fig. 2.**
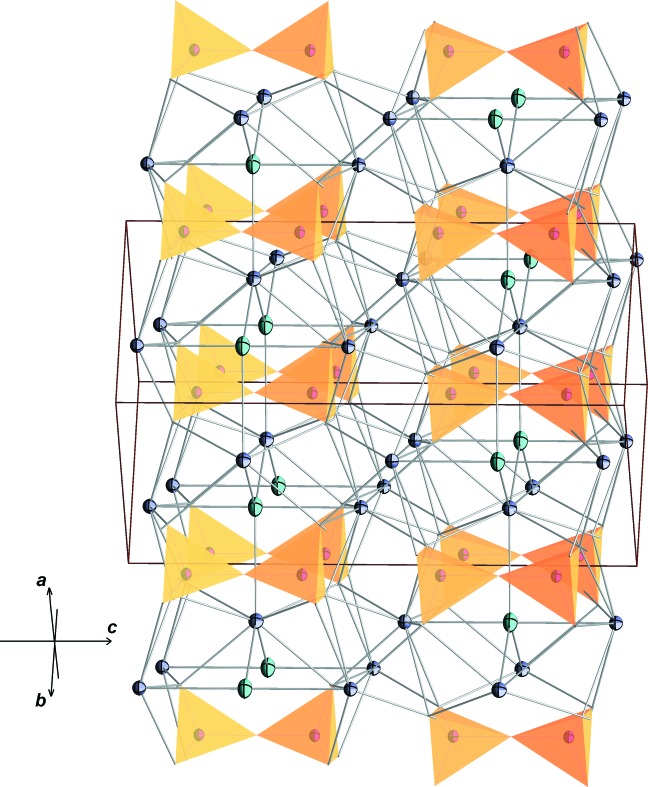
The crystal structure of Cs3(Mo2O7)Br in a projection approximately on (110). Cs atoms are represented as light-purple spheres and Br atoms as green spheres; MoO4 tetrahedra are given in yellow and orange. Ellipsoids are drawn at the 50% probability level.

## supplementary crystallographic information

### Comment

The structure of Cs_3_(Mo_2_O_7_)Br contains one symmetrically independent Mo^6+^ cation which is tetrahedrally coordinated by O atoms. Two (MoO_4_)^2-^ tetrahedra share a common O2 atom to form a [Mo_2_O_7_]^2-^ dimolybdate(VI) anion. The Mo—O2—Mo bond angle is linear and oriented along [001] (Fig. 1). In other dimolybdates(VI), this fragment differs from linearity and Mo—O—Mo bond angles range from 141.4° (Ce_2_(MoO_4_)_2_(Mo_2_O_7_); Fallon & Gatehouse, 1982) to 160.6° (Mg_2_Mo_2_O_7_; Stadnicka *et al.*, 1977). The corner linkage of tetrahedra is associated with bond-length distortions: the <Mo—O1> bond length is 1.725 (4) Å, whereas the <Mo—O2> bond-length is 1.8764 (7) Å; the O—Mo—O bond angles range from 108.34 (14)° (for <O1—Mo—O1>) to 110.58 (13)° (for <O1—Mo—O2>). The structure also contains two symmetrically independent Cs atoms and one Br atom. Cs1 is coordinated by nine O atoms and one Br atom, whereas Cs2 is coordinated by six O atoms and three Br atoms. The <Cs—O> bond lengths are in the range from 3.126 (4) Å to 3.239 (4) Å. The <Cs—Br> bond lengths are 3.4268 (6) Å and 3.6946 (3) Å. The [Mo_2_O_7_]^2-^ anions are linked by Cs2 atoms to form sheets running parallel to (001). The three-dimensional connectivity of the structure is provided by Cs1 atoms located in the interlayer (Fig. 2).

### Experimental

The title compound was prepared by the reaction of CsNO_3_ (0.192 g), MoO_3_ (0.146 g) and the ionic-liquid salt 1-ethyl-3-methylimidazolium bromide, [emim]Br (0.451 g). The mixture was heated to 453 K for 3 days in a teflon-lined steel autoclave with an internal volume of 20 ml. The obtained crystals were washed out with distilled water and dried in air at room temperature. A suitable colorless plate-shaped single-crystal was selected for X-ray structure analysis.

### Crystal data

**Table:**

Cs_3_(Mo_2_O_7_)Br	*D*_x_ = 4.445 Mg m^−^^3^
*M**_r_* = 782.52	Mo *K*α radiation, λ = 0.71073 Å
Hexagonal, *P*6_3_/*m**m**c*	Cell parameters from 5704 reflections
Hall symbol: -P 6c 2c	θ = 2.5–29.5°
*a* = 6.3993 (5) Å	µ = 14.77 mm^−^^1^
*c* = 16.4870 (15) Å	*T* = 293 K
*V* = 584.71 (8) Å^3^	Plate, colorless
*Z* = 2	0.15 × 0.15 × 0.05 mm
*F*(000) = 680	

### Data collection

**Table:**

Stoe IPDS-2 diffractometer	344 independent reflections
Radiation source: fine-focus sealed tube	338 reflections with *I* > 2σ(*I*)
graphite	*R*_int_ = 0.044
Detector resolution: 6.67 pixels mm^-1^	θ_max_ = 29.2°, θ_min_ = 2.5°
rotation method scans	*h* = −8→8
Absorption correction: integration (*X-RED* and *X-SHAPE*; Stoe & Cie, 2007)	*k* = −7→8
*T*_min_ = 0.153, *T*_max_ = 0.532	*l* = −21→22
5224 measured reflections	

### Refinement

**Table:**

Refinement on *F*^2^	Primary atom site location: structure-invariant direct methods
Least-squares matrix: full	Secondary atom site location: difference Fourier map
*R*[*F*^2^ > 2σ(*F*^2^)] = 0.025	*w* = 1/[σ^2^(*F*_o_^2^) + (0.0295*P*)^2^ + 2.906*P*] where *P* = (*F*_o_^2^ + 2*F*_c_^2^)/3
*wR*(*F*^2^) = 0.062	(Δ/σ)_max_ < 0.001
*S* = 1.18	Δρ_max_ = 0.60 e Å^−^^3^
344 reflections	Δρ_min_ = −1.21 e Å^−^^3^
20 parameters	Extinction correction: *SHELXL97* (Sheldrick, 2008), Fc^*^=kFc[1+0.001xFc^2^λ^3^/sin(2θ)]^-1/4^
0 restraints	Extinction coefficient: 0.0306 (16)

### Special details

**Table:**

Geometry. All e.s.d.'s (except the e.s.d. in the dihedral angle between two l.s. planes) are estimated using the full covariance matrix. The cell e.s.d.'s are taken into account individually in the estimation of e.s.d.'s in distances, angles and torsion angles; correlations between e.s.d.'s in cell parameters are only used when they are defined by crystal symmetry. An approximate (isotropic) treatment of cell e.s.d.'s is used for estimating e.s.d.'s involving l.s. planes.
Refinement. Refinement of *F*^2^ against ALL reflections. The weighted *R*-factor *wR* and goodness of fit *S* are based on *F*^2^, conventional *R*-factors *R* are based on *F*, with *F* set to zero for negative *F*^2^. The threshold expression of *F*^2^ > σ(*F*^2^) is used only for calculating *R*-factors(gt) *etc*. and is not relevant to the choice of reflections for refinement. *R*-factors based on *F*^2^ are statistically about twice as large as those based on *F*, and *R*- factors based on ALL data will be even larger.

### Fractional atomic coordinates and isotropic or equivalent isotropic displacement parameters (Å^2^)

**Table:**

	*x*	*y*	*z*	*U*_iso_*/*U*_eq_	
Cs1	1.3333	−0.3333	−0.04215 (3)	0.0227 (2)	
Cs2	0.6667	−0.6667	−0.2500	0.0265 (3)	
Mo	1.0000	0.0000	−0.13619 (4)	0.0166 (2)	
O1	0.8543 (4)	−0.2913 (7)	−0.0994 (2)	0.0274 (9)	
O2	1.0000	0.0000	−0.2500	0.029 (2)	
Br	1.3333	−0.3333	−0.2500	0.0365 (4)	

### Atomic displacement parameters (Å^2^)

**Table:**

	*U*^11^	*U*^22^	*U*^33^	*U*^12^	*U*^13^	*U*^23^
Cs1	0.0239 (3)	0.0239 (3)	0.0204 (3)	0.01194 (14)	0.000	0.000
Cs2	0.0274 (3)	0.0274 (3)	0.0246 (4)	0.01372 (17)	0.000	0.000
Mo	0.0183 (3)	0.0183 (3)	0.0130 (4)	0.00917 (14)	0.000	0.000
O1	0.0328 (18)	0.018 (2)	0.0265 (18)	0.0091 (10)	0.0023 (7)	0.0046 (15)
O2	0.040 (4)	0.040 (4)	0.008 (4)	0.0200 (19)	0.000	0.000
Br	0.0434 (6)	0.0434 (6)	0.0227 (7)	0.0217 (3)	0.000	0.000

### Geometric parameters (Å, °)

**Table:**

Cs1—O1^i^	3.126 (4)	Cs2—O2^xv^	3.6946 (3)
Cs1—O1^ii^	3.126 (4)	Cs2—O2^xiv^	3.6946 (3)
Cs1—O1^iii^	3.126 (4)	Cs2—O2	3.6946 (3)
Cs1—O1^iv^	3.3441 (12)	Cs2—Br^xvi^	3.6946 (3)
Cs1—O1^v^	3.3441 (12)	Mo—O1^iv^	1.725 (4)
Cs1—O1	3.3441 (12)	Mo—O1^xvii^	1.725 (4)
Cs1—O1^vi^	3.3441 (12)	Mo—O1	1.725 (4)
Cs1—O1^vii^	3.3441 (12)	Mo—O2	1.8764 (7)
Cs1—O1^viii^	3.3441 (12)	Mo—Cs1^xviii^	4.0067 (4)
Cs1—Br	3.4268 (6)	Mo—Cs1^xvi^	4.0067 (4)
Cs1—Cs1^ix^	3.9475 (5)	Mo—Cs2^xviii^	4.1438 (4)
Cs1—Cs1^iii^	3.9475 (5)	Mo—Cs2^xix^	4.1438 (4)
Cs2—O1^x^	3.239 (4)	O1—Cs1^iii^	3.126 (4)
Cs2—O1^v^	3.239 (4)	O1—Cs1^xvi^	3.3441 (12)
Cs2—O1^xi^	3.239 (4)	O2—Mo^xii^	1.8764 (7)
Cs2—O1	3.239 (4)	O2—Cs2^xviii^	3.6946 (3)
Cs2—O1^xii^	3.239 (4)	O2—Cs2^xix^	3.6946 (3)
Cs2—O1^xiii^	3.239 (4)	Br—Cs1^xii^	3.4268 (6)
Cs2—Br^xiv^	3.6946 (3)	Br—Cs2^xix^	3.6946 (3)
Cs2—Br	3.6946 (3)	Br—Cs2^vi^	3.6946 (3)
			
O1^i^—Cs1—O1^ii^	70.37 (12)	O1^v^—Cs2—O2^xv^	50.04 (7)
O1^i^—Cs1—O1^iii^	70.37 (12)	O1^xi^—Cs2—O2^xv^	108.73 (3)
O1^ii^—Cs1—O1^iii^	70.37 (12)	O1—Cs2—O2^xv^	108.73 (3)
O1^i^—Cs1—O1^iv^	68.67 (13)	O1^xii^—Cs2—O2^xv^	108.73 (3)
O1^ii^—Cs1—O1^iv^	104.90 (6)	O1^xiii^—Cs2—O2^xv^	108.73 (3)
O1^iii^—Cs1—O1^iv^	137.64 (3)	Br^xiv^—Cs2—O2^xv^	60.0
O1^i^—Cs1—O1^v^	104.90 (6)	Br—Cs2—O2^xv^	60.0
O1^ii^—Cs1—O1^v^	137.64 (3)	O1^x^—Cs2—O2^xiv^	108.73 (3)
O1^iii^—Cs1—O1^v^	68.67 (13)	O1^v^—Cs2—O2^xiv^	108.73 (3)
O1^iv^—Cs1—O1^v^	112.36 (6)	O1^xi^—Cs2—O2^xiv^	50.04 (7)
O1^i^—Cs1—O1	68.67 (13)	O1—Cs2—O2^xiv^	108.73 (3)
O1^ii^—Cs1—O1	137.64 (3)	O1^xii^—Cs2—O2^xiv^	108.73 (3)
O1^iii^—Cs1—O1	104.90 (6)	O1^xiii^—Cs2—O2^xiv^	50.04 (7)
O1^iv^—Cs1—O1	49.43 (14)	Br^xiv^—Cs2—O2^xiv^	60.0
O1^v^—Cs1—O1	65.19 (14)	Br—Cs2—O2^xiv^	180.0
O1^i^—Cs1—O1^vi^	137.64 (3)	O2^xv^—Cs2—O2^xiv^	120.0
O1^ii^—Cs1—O1^vi^	68.67 (13)	O1^x^—Cs2—O2	108.73 (3)
O1^iii^—Cs1—O1^vi^	104.90 (6)	O1^v^—Cs2—O2	108.73 (3)
O1^iv^—Cs1—O1^vi^	112.36 (6)	O1^xi^—Cs2—O2	108.73 (3)
O1^v^—Cs1—O1^vi^	112.36 (6)	O1—Cs2—O2	50.04 (7)
O1—Cs1—O1^vi^	146.20 (13)	O1^xii^—Cs2—O2	50.04 (7)
O1^i^—Cs1—O1^vii^	104.90 (6)	O1^xiii^—Cs2—O2	108.73 (3)
O1^ii^—Cs1—O1^vii^	68.67 (13)	Br^xiv^—Cs2—O2	180.0
O1^iii^—Cs1—O1^vii^	137.64 (3)	Br—Cs2—O2	60.0
O1^iv^—Cs1—O1^vii^	65.19 (14)	O2^xv^—Cs2—O2	120.0
O1^v^—Cs1—O1^vii^	146.20 (13)	O2^xiv^—Cs2—O2	120.0
O1—Cs1—O1^vii^	112.36 (6)	O1^x^—Cs2—Br^xvi^	129.96 (7)
O1^vi^—Cs1—O1^vii^	49.43 (14)	O1^v^—Cs2—Br^xvi^	129.96 (7)
O1^i^—Cs1—O1^viii^	137.64 (3)	O1^xi^—Cs2—Br^xvi^	71.27 (3)
O1^ii^—Cs1—O1^viii^	104.90 (6)	O1—Cs2—Br^xvi^	71.27 (3)
O1^iii^—Cs1—O1^viii^	68.67 (13)	O1^xii^—Cs2—Br^xvi^	71.27 (3)
O1^iv^—Cs1—O1^viii^	146.20 (13)	O1^xiii^—Cs2—Br^xvi^	71.27 (3)
O1^v^—Cs1—O1^viii^	49.43 (14)	Br^xiv^—Cs2—Br^xvi^	120.0
O1—Cs1—O1^viii^	112.36 (6)	Br—Cs2—Br^xvi^	120.0
O1^vi^—Cs1—O1^viii^	65.19 (14)	O2^xv^—Cs2—Br^xvi^	180.0
O1^vii^—Cs1—O1^viii^	112.36 (6)	O2^xiv^—Cs2—Br^xvi^	60.0
O1^i^—Cs1—Br	138.29 (8)	O2—Cs2—Br^xvi^	60.0
O1^ii^—Cs1—Br	138.29 (8)	O1^iv^—Mo—O1^xvii^	108.34 (14)
O1^iii^—Cs1—Br	138.29 (8)	O1^iv^—Mo—O1	108.34 (14)
O1^iv^—Cs1—Br	73.60 (6)	O1^xvii^—Mo—O1	108.34 (14)
O1^v^—Cs1—Br	73.60 (6)	O1^iv^—Mo—O2	110.58 (13)
O1—Cs1—Br	73.60 (6)	O1^xvii^—Mo—O2	110.58 (13)
O1^vi^—Cs1—Br	73.60 (6)	O1—Mo—O2	110.58 (13)
O1^vii^—Cs1—Br	73.60 (6)	Mo—O1—Cs1^iii^	152.3 (2)
O1^viii^—Cs1—Br	73.60 (6)	Mo—O1—Cs2	109.37 (16)
O1^x^—Cs2—O1^v^	100.09 (14)	Cs1^iii^—O1—Cs2	98.33 (11)
O1^x^—Cs2—O1^xi^	67.58 (11)	Mo—O1—Cs1	99.46 (8)
O1^v^—Cs2—O1^xi^	142.54 (6)	Cs1^iii^—O1—Cs1	75.10 (6)
O1^x^—Cs2—O1	142.54 (6)	Cs2—O1—Cs1	99.88 (7)
O1^v^—Cs2—O1	67.58 (11)	Mo—O1—Cs1^xvi^	99.46 (8)
O1^xi^—Cs2—O1	142.54 (6)	Cs1^iii^—O1—Cs1^xvi^	75.10 (6)
O1^x^—Cs2—O1^xii^	67.58 (11)	Cs2—O1—Cs1^xvi^	99.88 (7)
O1^v^—Cs2—O1^xii^	142.54 (6)	Cs1—O1—Cs1^xvi^	146.20 (13)
O1^xi^—Cs2—O1^xii^	67.58 (11)	Mo^xii^—O2—Mo	180.0
O1—Cs2—O1^xii^	100.09 (14)	Mo^xii^—O2—Cs2^xviii^	90.0
O1^x^—Cs2—O1^xiii^	142.54 (6)	Mo—O2—Cs2^xviii^	90.0
O1^v^—Cs2—O1^xiii^	67.58 (11)	Mo^xii^—O2—Cs2	90.0
O1^xi^—Cs2—O1^xiii^	100.09 (14)	Mo—O2—Cs2	90.0
O1—Cs2—O1^xiii^	67.58 (11)	Cs2^xviii^—O2—Cs2	120.0
O1^xii^—Cs2—O1^xiii^	142.54 (6)	Mo^xii^—O2—Cs2^xix^	90.0
O1^x^—Cs2—Br^xiv^	71.27 (3)	Mo—O2—Cs2^xix^	90.0
O1^v^—Cs2—Br^xiv^	71.27 (3)	Cs2^xviii^—O2—Cs2^xix^	120.0
O1^xi^—Cs2—Br^xiv^	71.27 (3)	Cs2—O2—Cs2^xix^	120.0
O1—Cs2—Br^xiv^	129.96 (7)	Cs1^xii^—Br—Cs1	180.0
O1^xii^—Cs2—Br^xiv^	129.96 (7)	Cs1^xii^—Br—Cs2^xix^	90.0
O1^xiii^—Cs2—Br^xiv^	71.27 (3)	Cs1—Br—Cs2^xix^	90.0
O1^x^—Cs2—Br	71.27 (3)	Cs1^xii^—Br—Cs2	90.0
O1^v^—Cs2—Br	71.27 (3)	Cs1—Br—Cs2	90.0
O1^xi^—Cs2—Br	129.96 (7)	Cs2^xix^—Br—Cs2	120.0
O1—Cs2—Br	71.27 (3)	Cs1^xii^—Br—Cs2^vi^	90.0
O1^xii^—Cs2—Br	71.27 (3)	Cs1—Br—Cs2^vi^	90.0
O1^xiii^—Cs2—Br	129.96 (7)	Cs2^xix^—Br—Cs2^vi^	120.0
Br^xiv^—Cs2—Br	120.0	Cs2—Br—Cs2^vi^	120.0
O1^x^—Cs2—O2^xv^	50.04 (7)		

Symmetry codes: (i) *x*−*y*, *x*−1, −*z*; (ii) *y*+2, −*x*+*y*+1, −*z*; (iii) −*x*+2, −*y*−1, −*z*; (iv) −*y*+1, *x*−*y*−1, *z*; (v) −*x*+*y*+2, −*x*, *z*; (vi) *x*+1, *y*, *z*; (vii) −*x*+*y*+3, −*x*+1, *z*; (viii) −*y*+1, *x*−*y*−2, *z*; (ix) −*x*+3, −*y*, −*z*; (x) −*x*+*y*+2, −*x*, −*z*−1/2; (xi) −*y*, *x*−*y*−2, −*z*−1/2; (xii) *x*, *y*, −*z*−1/2; (xiii) −*y*, *x*−*y*−2, *z*; (xiv) *x*−1, *y*−1, *z*; (xv) *x*, *y*−1, *z*; (xvi) *x*−1, *y*, *z*; (xvii) −*x*+*y*+2, −*x*+1, *z*; (xviii) *x*, *y*+1, *z*; (xix) *x*+1, *y*+1, *z*.
